# Can Proteomics Play a Significant Role in the Identification of Biomarkers for Alpha1-Antitrypsin Deficiency?

**DOI:** 10.3390/ijms26115085

**Published:** 2025-05-26

**Authors:** Maria Antonietta Grignano, Maura D’Amato, Marilena Gregorini, Teresa Rampino, Paolo Iadarola, Simona Viglio

**Affiliations:** 1 Unit of Nephrology, Dialysis and Transplantation, IRCCS Policlinico San Matteo Foundation, 27100 Pavia, Italy; ma.grignano@smatteo.pv.it (M.A.G.); M.Gregorini@smatteo.pv.it (M.G.); T.Rampino@smatteo.pv.it (T.R.); 2 Department of Molecular Medicine, University of Pavia, 27100 Pavia, Italy; maura.damato90@gmail.com (M.D.); simona.viglio@unipv.it (S.V.); 3 Department of Internal Medicine and Therapeutics, University of Pavia, 27100 Pavia, Italy; 4 Department of Biology and Biotechnologies “L. Spallanzani”, University of Pavia, 27100 Pavia, Italy; 5 Lung Transplantation Unit, IRCCS Policlinico San Matteo Foundation, 27100 Pavia, Italy

**Keywords:** α1-antitrypsin, α1-antitrypsin deficiency, COPD, LC-MS/MS

## Abstract

Alpha-1 antitrypsin deficiency (AATD) is a common genetic disorder that can manifest in a broad spectrum of clinical symptoms, ranging from asymptomatic cases to severe, progressive systemic diseases, primarily affecting the lungs and liver. Despite its prevalence, AATD is often perceived as a rare condition, which can lead to a lack of awareness among primary care physicians and even some respiratory specialists. This misconception may result in missed opportunities for diagnosis, particularly in mild or asymptomatic patients. Consequently, it is vital for healthcare providers to familiarize themselves with the various presentations, diagnostic techniques, and management strategies for AATD. This review explores the current understanding of AATD, emphasizing the valuable role of liquid chromatography-mass spectrometry in identifying biomarkers that could enhance early diagnosis and help predict disease outcomes. As knowledge about the complexities of AATD continues to grow, physicians may begin to view the disorder not as a fatal pathology, but as a treatable inherited condition with the potential for improved management.

## 1. Introduction

Alpha1-antitrypsin (AAT) is a liver-produced single-chain glycoprotein, synthesized as a 418-amino-acid precursor, that enters the bloodstream to protect tissues from the damaging effects of harmful proteases [1,2]. AAT is particularly specific for human neutrophil elastase (HNE), released by neutrophils during inflammation, but also inhibits other proteases, including metalloproteases, cathepsin G, proteinase 3, and cysteine and aspartic proteases [3]. Encoded by the *SERPINA1* gene, also called the Pi gene, located on chromosome 14 (14q31–32.3), AAT belongs to the serpin superfamily.

Currently, over two hundred *SERPINA1* variants have been identified, most resulting from point mutations in the gene sequence that lead to amino acid substitutions [4,5]. Several substitutions may affect the electrophoretic mobility of the protein, thus allowing these variants to be detected by isoelectric focusing (IEF). They are labeled A to Z if their electrophoretic mobility is faster or slower, respectively, compared to that of the most common variant, labeled M [4]. The M variant (normal variant) is not pathogenic, and individuals homozygous for this variant (referred to as PiMM individuals) show normal AAT function and serum levels. The most frequent pathogenic AAT variants are the S (c.863A>T; p.Glu288Val) and Z (c.1096G>T; p.Glu366Lys) variants, which express, respectively, approximately 50–60% and 10–20% of normal AAT levels. The MM, MS, MZ, SS, SZ and ZZ protein phenotypes account for >99% of all variants [6].

**Figure 1:**
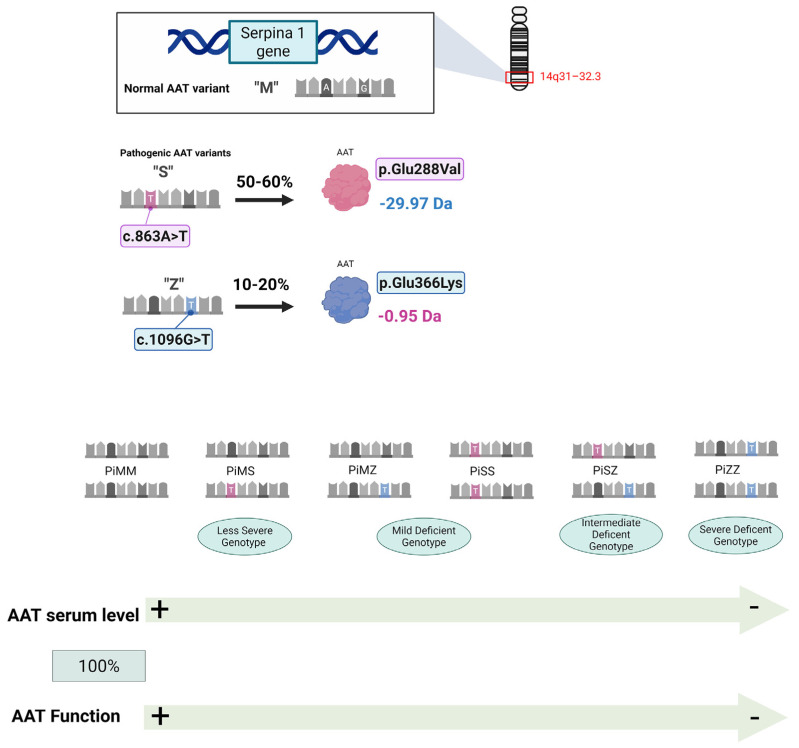
The diagram displays, from top to bottom, the gene sequence of the normal AAT variant ‘M’ (shown in the box); the point mutations that produce the ‘S’ and ‘Z’ variants; the resulting amino acid substitutions; and the corresponding mass changes in the tryptic peptides containing these substitutions. The most common AAT variants are shown below. Created in BioRender. Rampino, t. (2025) https://BioRender.com/nvcvxfl (accessed on 21 April 2025).

A diagram displaying the gene sequence of the normal AAT variant ‘M’, the point mutations producing the ‘S’ and ‘Z’ variants, the resulting amino acid substitutions, and the corresponding mass changes in the tryptic peptides containing these substitutions is shown in Figure 1.

The Z-AAT mutant (and other rare pathogenic variants) predisposes to liver disease and/or to a lung disorder known as alpha1-antitrypsin deficiency (AATD). If liver disease is caused by the accumulation of this variant as polymeric chains in hepatocytes [7], the low AAT levels in blood and lungs result in an unbalance between proteinases and anti-proteinases [8,9]. This can cause structural changes in lung parenchyma, leading to lung conditions such as bronchiectasis, chronic obstructive pulmonary disease (COPD) and emphysema [10,11,12,13].

AATD is a genetically inherited disorder usually diagnosed through serum protein concentration measurement and the identification of allelic variants via phenotyping or allele-specific genotyping [14,15,16,17,18]. However, the intrinsic complexity of the disorder and the variety of genetic forms result in significant clinical variability, even among individuals with the same genetic form and similar AAT blood levels.

Thus, AATD often goes unrecognized/underdiagnosed in clinical practice [19,20], which is concerning since unchecked deficiency leads to ongoing lung proteolysis, elastin degradation, and alveolar damage. It is well established that individuals diagnosed with AATD before the onset of pulmonary symptoms typically have better outcomes than those diagnosed at later stages when respiratory illness is already in progress. Mass Spectrometry (MS), a powerful analytical tool, is emerging in this context as a promising approach for identifying and quantifying proteins and peptides involved in AATD, potentially improving early detection of disease biomarkers and patient outcomes [21].

This report aims to update on emerging proteomic techniques for the detection of AATD biomarkers and of common deficiency alleles, S and Z, linked with AATD.

## 2. Introducing Alpha1-Antitrypsin Deficiency (AATD) and Potential Treatments

**Figure 2:**
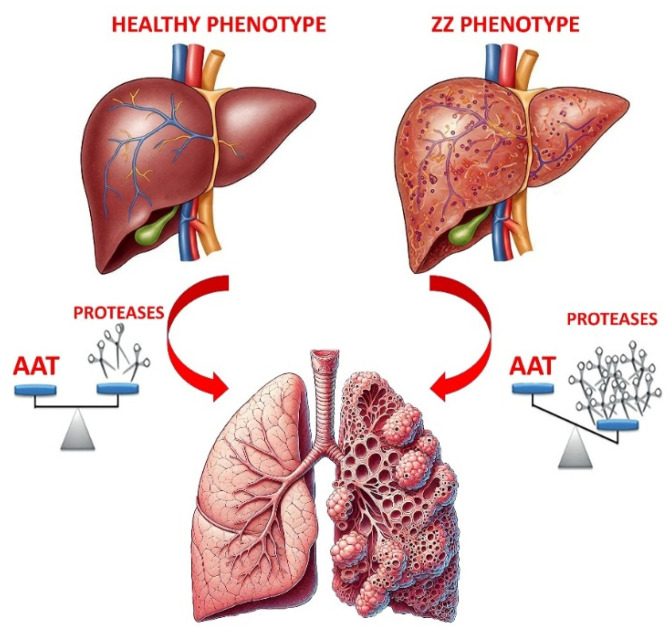
The image compares two different lung conditions. On the left, a healthy lung is shown, marked by a balanced ratio between protease activity and AAT levels. In contrast, the right side depicts lung tissue damage caused by an imbalance—where proteases are significantly elevated relative to AAT.

As noted, AATD is an inherited genetic disorder leading to low circulating levels of AAT, the primary systemic antiproteinase that protects the lungs. Reduced AAT production raises the risk of serious health issues, from liver damage to respiratory symptoms (see Figure 2).

Liver disease severity varies widely among AATD patients, with liver transplantation being the only available specific treatment. Respiratory disorders are typically chronic cough, shortness of breath and wheezing, resembling emphysema. AATD has no cure, but available treatments, while not being able to reverse lung damage, can offer symptom relief and prevent further harm. Augmentation therapy is the primary treatment specific for lung disease, consisting in the raise of AAT levels by infusing directly into bloodstream purified AAT from healthy blood donors [22]. This therapy, typically administered weekly, is reserved for individuals with the lowest AAT levels. Although it aims to reduce lung density loss thus slowing disease progression, its efficacy remains debated [23]. Additional treatments, such as bronchodilators and oxygen therapy reduce inflammation and open airways thus making breathing easier. Other potential strategies, including gene therapy and the use of induced pluripotent stem cells, are under investigation [16].

## 3. Proteomics and Biomarker Discovery

Since early AATD symptoms are often mild and overlap with those of other illnesses, the diagnosis of this disorder may be delayed. Testing for AATD patients with liver symptoms or those diagnosed with COPD could improve the rate of early diagnosis [19]. A significant advancement in diagnostic and therapeutic technologies may lead to the identification in body fluids or tissues of molecular markers that can efficiently and accurately detect early stages of disorders. Of great interest could also be biomarkers whose level responds to medical treatments. Their use in clinical trials allows monitoring the progression of patient condition and understanding changes in patient’s clinical outcome [24].

**Figure 3:**
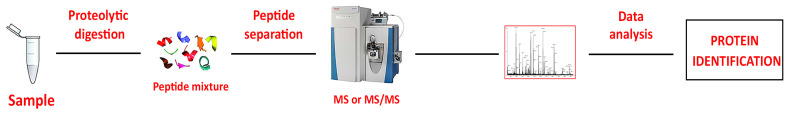
“Bottom up” approach for proteomic studies.

Proteomic analysis using liquid chromatography coupled with tandem mass spectrometry (LC-MS/MS) [25] is currently the most widely used method for biomarker discovery. Its high sensitivity and specificity have enabled the identification of numerous potential protein biomarkers for various diseases [25]. The rationale for this choice is that all disorders are characterized by molecular alterations that often precede clinical manifestations by a considerable time. Thus, the focus of most clinical proteomics studies is the capture of differences in relative protein abundance under different conditions. The so-called “Bottom-up” methods (Figure 3) are widely used in proteomics for protein identification. In this approach, all proteins in a sample are digested with a protease, typically trypsin, generating a mixture of peptides. These peptides are then separated via liquid chromatography (LC), and their sequences are acquired using MS/MS. Subsequent analysis with advanced software tools for database searching of MS/MS data enables accurate identification and quantification of the proteins from which the peptides originated [26].

Current proteomic technologies have led to significant advances in our understanding of the biological mechanisms underlying health and disease, paving the way for improved patient care.

## 4. Proteomic Analysis of AATD

### 4.1. Application to Lung and Liver Diseases

AATD is the most common genetic cause of emphysema and is characterized by unexplained phenotypic heterogeneity among affected individuals [6]. Due to the diversity of pathogenic mutations, there is limited guidance on monitoring and treating patients when the clinical significance of variants is unknown. To explore the origins of this heterogeneity, Serban et al. [27] analyzed plasma/serum samples from 5924 subjects across four independent cohorts, including a large COPD population and three AATD groups. Using the SomaScan V4.0 platform, a multiplex proteomic technology recognized for its reproducibility [28], the authors aimed to identify new plasma biomarkers for AATD-associated emphysema correlated with clinical outcomes. With the support of different statistical methods, their analysis revealed highly sensitive and specific biomarkers that identified severe- (PiZZ), intermediate- (PiSZ), and mild-deficient (PiMZ) genotypes. Detection of the less severe genotype (PiMS) was lower due to the similarity of AAT levels with those of the normal variant PiMM. They also uncovered unique and shared plasma biomarkers between AATD and COPD patients and identified proteins associated with emphysema in both groups. Specifically, PiZZ patients displayed biomarkers linked to diffusing capacity for carbon monoxide (DLCO) and emphysema. Additionally, the results confirmed the elevation of AAT to near-normal levels in PiZZ individuals undergoing augmentation therapy. In summary, the study utilized a large, diverse cohort and advanced proteomic technology to identify both unique and shared biomarkers associated with emphysema in AATD and COPD. While the findings offer valuable insights, considerations regarding technological limitations and potential confounding factors are essential for interpreting the results and guiding future research. In fact, although the use of the SomaScan V4.0 aptamer-based proteomic platform enabled broad protein quantification, platform-specific biases and variable aptamer affinities may affect accuracy. Additionally, potential confounding factors such as cohort heterogeneity, smoking status, and augmentation therapy, along with the cross-sectional study design, may limit causal inference.

Beiko et al. [29] conducted the quantitative lung computed tomography (CT) unmasking emphysema progression in AATD study (QUANTUM-1) to identify proteomic signatures associated with emphysema progression in patients with severe AATD but normal forced expiratory volume in 1 s (FEV1). The aim of this research was the identification of serum proteins that correlated with CT density decline. The study initially enrolled 49 adults with severe AATD and post-bronchodilator FEV_1_ ≥ 80% predicted. However, only 31 participants had complete proteomic and lung densitometry data and were included in the final analysis. The analysis revealed four serum proteins (C-reactive protein, CRP; adipocyte fatty acid-binding protein, AFBP; leptin and tissue plasminogen activator, tPA) correlated with baseline emphysema. After adjustments (e.g., for age and sex), all but leptin were linked to body mass index (BMI) and also to emphysema progression, making these proteins potential therapeutic targets for COPD. When evaluating these findings, it is essential to acknowledge methodological limitations that may influence their reliability. The use of the Myriad Discovery MAP^®^ multiplex immunoassay enabled broad proteomic coverage but is inherently limited by factors such as variable antibody specificity, potential matrix effects, and inter-assay variability. Moreover, the small sample size and cross-sectional design reduce statistical power and prevent causal inference. Notably, several identified biomarkers showed strong associations with BMI, raising concerns about confounding, particularly given the inclusion of participants with severe obesity (BMI > 40), which may independently affect lung density. These considerations underscore the need for cautious interpretation and independent validation in larger, longitudinal studies.

Liver disease in AATD follows a biphasic pattern, peaking in early childhood and adulthood. Karatas et al. [30] applied proteomic analysis to formalin-fixed paraffin-embedded (FFPE) liver tissues to investigate factors underlying adult AATD-related liver disease. They compared samples from eight PiZZ patients (four pediatric, four adults) who underwent liver transplantation with those from healthy controls (three pediatric, four adults). Proteins differentially expressed between pediatric and adult patients were obtained by laser microdissection of hepatocytes and revealed by MS. Among 65 proteins upregulated exclusively in adult PiZZ samples, four members of the protein disulfide isomerase (PDI) family (PDIA4, PDIA3, P4HB, and TXNDC5) were identified. Notably, PDIA4 appeared to have a good therapeutic target, since its inhibition by cysteamine reduced Z-aggregate formation and its silencing lowered oxidative stress, a feature of AATD-mediated liver disease. This suggested that PDI inhibition is a potential therapeutic approach for AATD treatment, though several methodological considerations warrant discussion. The sample size of the study was limited due to the scarcity of well-characterized human liver specimens, potentially impacting the robustness and generalizability of findings. The MS-based proteomic analysis, though powerful, is subject to inherent biases including preferential detection of abundant proteins and challenges in capturing low-abundance or hydrophobic membrane proteins. Additionally, variability in tissue quality, patient heterogeneity, and sample processing introduces potential confounders that might influence the observed proteomic profiles. Although matched controls were employed to mitigate these effects, residual confounding cannot be excluded. Future studies with larger cohorts and complementary technologies could help validate and extend these findings.

Ohlmeier et al. [31] applied two-dimensional difference gel electrophoresis (DIGE) and MS to perform a proteomic study on lung tissues from eight AATD and nine idiopathic pulmonary fibrosis (IPF) patients, along with nonsmokers (*n* = 9), healthy smokers (*n* = 9), and smokers with COPD at varying severity (*n* = 16). Their study revealed disease-specific protein changes in AATD, IPF, and COPD, with findings transferred from lung tissue to more accessible sputum and plasma samples. The discovery that transglutaminase 2 (TGM2) was increased across all sample types supports its potential as a target for diagnosing and treating AATD-associated COPD. While the findings contribute to the understanding of COPD pathogenesis, several methodological limitations should be acknowledged. The relatively small sample size, reflecting the challenge of obtaining well-characterized lung tissue, may limit statistical power and the broader applicability of the results. The study employed 2D-DIGE and mass spectrometry, established proteomic techniques that nonetheless have inherent limitations, including reduced sensitivity for low-abundance, hydrophobic, or high-molecular-weight proteins. This potentially leads to a biased representation of the proteome. Furthermore, variability in tissue composition due to inflammation or fibrosis may have affected protein extraction and detection. Confounding factors such as differences in smoking history, medication use, and the severity of lung damage among participants could influence the proteomic profiles. Although the study used matched controls to mitigate some of these effects, residual confounding is likely. Nonetheless, the identification of elevated transglutaminase 2 in stable COPD provides a meaningful lead for further research, particularly in studies with larger cohorts and complementary molecular approaches.

Building on the observation that patients without AATD typically develop COPD later in life than those with the condition, Murphy et al. [32] explored whether the absence of plasma AAT could influence the neutrophil characteristics of COPD phenotypes, whether or not they are associated with AATD. Given the established binding of AAT to neutrophil plasma membranes, specifically within membrane lipid rafts [33], an intriguing question arises: could exogenous AAT administered through augmentation therapy to AATD-COPD patients bind to circulating AATD neutrophils, potentially shifting their phenotype towards that observed in non-AATD-COPD? To explore the potential consequences of altered membrane protein expression on neutrophil function and to assess the impact of AAT augmentation therapy, the authors compared the plasma membrane proteomes of neutrophils from individuals with AATD-COPD (*n* = 25) to those from non-AATD-COPD patients (*n* = 7). The focus on the neutrophil plasma membrane stems from its role as the interface between the cell and its environment, which plays a crucial role in determining how a cell responds to stimuli. A label-free MS/MS proteomic analysis of plasma membranes from neutrophils of AATD- and non-AATD-COPD patients identified an average of 860 proteins. Among the 15 proteins that were differentially expressed between the two groups, 8 (including myeloperoxidase, MPO, and bactericidal/permeability increasing protein, BPI) were found to be more abundant in the plasma membrane fractions of AATD-COPD patients compared to non-AATD-COPD patients. These results suggest that AAT augmentation therapy influences the membrane proteome by altering the levels of membrane-associated proteins in circulating neutrophils of AATD-COPD patients, in contrast to their non-AATD-COPD counterparts. The study offered important mechanistic insights but was limited by a moderate sample size, which may affect generalizability. The use of mass spectrometry and membrane-enrichment techniques introduced typical proteomic biases, such as underrepresentation of low-abundance and hydrophobic proteins. Confounding factors, as patient variability in inflammation levels, treatment duration, and other medications, were partially controlled through paired analyses, but residual confounding remains. Despite these limitations, the findings support a broader immunomodulatory role for AAT therapy.

To overcome the limitations of current AATD diagnostic tools, Kemp et al. [21] developed proteotyping, a qualitative proteomic approach referred to as an alternative for detecting the most common alleles, S and Z, associated with this disorder. This technique involves trypsin-mediated peptide generation followed by LC-MS/MS analysis, enabling the detection of the S and Z alleles. The mutations associated with these alleles cause amino acid changes, leading to differences in the sequence and mass of peptides involved, compared to those from wild-type AAT (M alleles) (see Figure 1). The ability of MS to detect the mass differences between S/Z peptides and non-S/non-Z peptides enables the easy identification of the mutations. By combining the analysis of peptide patterns with AAT quantification through immunoassay, this approach provides an accurate assessment of deficiency alleles in most patients. While the authors present a robust and technically detailed LC-MS/MS method for AAT proteotyping, several limitations warrant considerations. First, as a methodological study, it was conducted on a limited number of samples (*n* = 5), which, while sufficient for technical validation, does not account for the biological variability encountered in clinical settings. The method’s performance across diverse patient cohorts, A1AT genotypes, and inflammatory or pathological conditions remains to be evaluated. Moreover, the method may be subject to technical biases common to targeted mass spectrometry, including peptide selection bias, incomplete digestion, and difficulty detecting low-abundance or heavily modified proteoforms. Finally, while the protocol shows promise for clinical translation, it has not yet been benchmarked against standard diagnostic workflows or assessed for clinical utility in large-scale studies. Future research should aim to validate the method in broader, clinically relevant cohorts.

A qualitative proteome profiling of exhaled breath condensate (EBC) from 15 COPD patients without emphysema, 23 individuals with pulmonary emphysema linked to AATD, 25 non-smokers (NS), and 10 healthy smokers (HS) was conducted by Fumagalli et al. [34], with the goal of determining whether protein patterns could reflect the distinct lung conditions across the different groups. LC-MS/MS analysis revealed a “fingerprint” of proteins in the EBC, including several inflammatory cytokines, type I and II cytokeratins, two isoforms of surfactant protein A (SP-A), calgranulins A and B, and AAT. Since all proteins found in the COPD and AATD groups were also present in the NS/HS control group, these findings could serve as a foundation for future quantitative studies aimed at identifying specific proteins that may differentiate healthy individuals from AATD patients or monitor effects of therapeutic interventions. While EBC is a non-invasive sample type, its low protein content and variability pose analytical challenges. The modest sample size limits statistical power, and the use of LC-MS/MS without protein enrichment may have biased detection toward abundant proteins. Confounding factors such as smoking status, medication use, and airway variability were only partially addressed. Despite these limitations, the study highlights the potential of EBC proteomics for biomarker discovery in respiratory disease.

The results obtained by the same research group from metabolomic analysis of EBC in patients with moderate and severe emphysema AATD-related (*n* = 11) compared to healthy individuals (*n* = 11) were notably more intriguing [35]. Nuclear magnetic resonance (NMR) analysis produced distinct profiles, revealing both qualitative and quantitative differences between these homogeneous patient and control groups. Among the metabolites that most effectively distinguished patients from controls, acetoin, propionate, acetate, and propane-1,2-diol exhibited the greatest discrepancies. The clear differentiation between the two groups, based on their metabolite content, was confirmed through univariate and multivariate statistical analyses. Using the MetaboAnalyst 3.0 platform to explore the relationships among metabolites, it was found that pyruvate metabolism is the most prominently involved pathway, with most metabolites originating from pyruvate. The ability of NMR to differentiate between the two groups of AATD patients and from healthy controls based on the metabolic fingerprints of their EBC highlights its significant potential for clinical application in this area. However, while this study provides preliminary metabolic insight into AATD, its results require confirmation in larger, better-controlled cohorts using complementary technologies. In fact, ^1^H NMR spectroscopy, while highly reproducible and non-destructive, is less sensitive than mass spectrometry, potentially missing low-abundance metabolites. This limits the breadth of detectable metabolic alterations and introduces bias toward more concentrated species.

A schematic summary of all articles commented above is shown in Table 1.

### 4.2. Limitations of Current Proteomic Studies

Although proteomic research in AATD has made notable strides, some limitations continue to hinder the translation of these findings into clinical practice. First, cohort heterogeneity remains a major obstacle. Several studies involve patients with varying genotypes, disease stages, and comorbid conditions, which reduces both the comparability of results and their broader applicability. Second, the absence of longitudinal validation makes it difficult to evaluate the stability of biomarkers over time or their effectiveness in monitoring disease progression. Third, functional assays are rarely incorporated, leaving the biological significance of many identified proteins unclear and limiting mechanistic insights. Finally, the lack of external validation in independent cohorts raises concerns about the robustness and reproducibility of proposed biomarkers. Overcoming these challenges is crucial to enhancing the clinical relevance of proteomic strategies in AATD.

### 4.3. Unmet Clinical and Research Needs in AATD

Despite the advances in understanding and managing AATD, several important clinical and research needs remain unmet. Clinically, key challenges include delayed or missed diagnosis, limited treatment options, gaps in the management of liver disease, and difficulties in addressing pediatric cases. From a research perspective, there is a pressing need to better understand disease heterogeneity, identify reliable biomarkers for diagnosis and monitoring, develop novel therapies, and establish improved animal and cellular models. Table 2 provides a comprehensive overview of these unmet needs.

## 5. Is Proteomics Still in the Early Stages of Research on AATD?

AATD has traditionally been regarded as a rare disease predominantly affecting Caucasians of northern European descent. However, recent research indicates that it is not as rare as previously thought, especially when compared to other genetic disorders, and is not limited to the Caucasian population [36]. With a prevalence of approximately 1 in 2000 to 5000 live births and a global distribution across all major racial groups, the carriers of deficiency alleles are millions. As a result, AATD may be one of the most common single-gene genetic disorders, leading to various clinical manifestations, primarily impacting the lungs and liver [37,38]. However, at the onset, these manifestations can be challenging to interpret, as they include asymptomatic or mild/moderate conditions which progress to more severe, debilitating systemic issues. Furthermore, disease progression often spans long periods and varies due to genetic and phenotypic differences, complicating both care decisions and the development of new treatments. Thus, early diagnosis is essential, allowing clinicians to initiate treatment at the earliest opportunity.

Assuming that proteomics can be applied to any disorder without limitations and is significantly advancing our understanding of various diseases, it seems reasonable to believe it could also be extremely valuable in studying AATD. However, given the limited number of publications, it appears that proteomics is still in its early stages in this field. If this is the case, it is still unclear why this platform has been underutilized in studying the disorder. One might question whether the reasons mentioned above could explain the low utilization of proteomics. Indeed, if AATD is continued to be regarded as a “rare” disease, like to many other rare conditions, it tends to be overlooked in research. The absence of a definitive cure, the availability of only one approved therapy, and the lack of clinical tools and validated biomarkers all support this hypothesis. However, there is progress happening in this field. While few in number, it is important to highlight some recent studies using a proteomic approach that expand on existing data.

A notable example is the study by Park et al. [39], who identified, by using commercially available enzyme-linked immunosorbent assay (ELISA) kits, club cell protein-16 (CC16) as a potential biomarker for lung function and disease progression in PiZZ patients. The recent proteomic research of Spittle et al. [40] further supports these findings revealing that elevated serum levels of CC16 are associated with a significant reduction in the transfer factor for carbon monoxide (TLCO). The unique pattern observed in PiZZ patients, compared to typical COPD cases, suggested that PiZZ individuals possess distinct characteristics that deserve more attention. The authors also highlighted the value of proteomics in validating these findings and identifying additional potential biomarkers for AATD. A very recent report by Moll et al. [41] further affirms the considerable potential of this platform within the AATD research community in identifying biomarkers crucial to this disorder. Also, this study can be seen as an extension and the validation of the previous data from Serban et al. [27] on plasma biomarkers for AATD-associated emphysema. In their study, the authors analyzed a large cohort of individuals from the COPD Gene (PiM) and the AAT Genetic Modifier Study (PiZZ), identifying 16 blood proteins linked to airflow obstruction in both groups. Of these proteins, 14 were highly expressed in the lungs, and a network-based enrichment analysis revealed alterations in immune system function, changes in cytokine and interleukin signaling, and in the regulation of matrix metalloproteinases. These findings could enhance our understanding of the pathogenesis of airflow obstruction and inform potential therapeutic strategies for individuals with severe AATD.

The previously cited work of Kemp et al. [21] also builds upon earlier studies [42,43], offering a relevant breakthrough in the field. It introduces an LC-MS/MS method that minimizes offline purification and reduces the labor involved in sample preparation, while also demonstrating potential in identifying correlations between allele concentrations and the development or severity of clinical symptoms. This approach enables allele-specific quantification in heterozygous patients, providing valuable biological and clinical insights into AAT function and deficiency. This work clearly demonstrates the significant role that proteomics plays in advancing the quantification of AAT allele protein expression in the serum of heterozygous individuals, identifying the most prevalent alleles, S and Z, associated with the disorder.

### 5.1. Integrative Multi-Omics Approaches for Predictive Modeling of AATD

It has been previously observed that symptoms and disease severity in patients with AATD exhibit significant variability. This heterogeneity is driven by a combination of individual patient characteristics and a broad range of environmental and genetic risk factors. Effectively addressing this complexity requires advanced technologies capable of capturing each patient’s unique molecular profile. Although proteomics alone has provided valuable insights, as discussed earlier, the multifactorial nature of AATD suggests that no single omics layer can fully elucidate the mechanisms involved in its onset and progression. Therefore, integrating multiple omics approaches is expected to enhance research outcomes and support a more comprehensive understanding of the disease. Combining proteomics with other omics layers, such as genomics, transcriptomics, and metabolomics, holds promise for uncovering the underlying biological pathways of AATD, ultimately contributing to improved patient stratification and the development of personalized therapeutic strategies. However, the application of integrative omics in AATD research remains limited. While proteomics has been extensively explored, only a few studies have investigated the potential of other omics approaches. For instance, metabolomics has been applied to exhaled breath condensate (EBC) from two groups of AATD patients with moderate and severe emphysema [35], and transcriptomics has been used to analyze liver tissue from AATD patients with and without lung disease [44]. Notably, one study integrated lipidomics and transcriptomics to compare liver organoids derived from healthy individuals and PiZZ patients [45].

### 5.2. Clinical Translation of Proteomic Biomarkers: Validation and Implementation

Translating proteomic biomarkers into clinical practice requires a rigorous validation process to ensure their reliability and clinical utility. This process encompasses several critical steps, including both analytical and clinical validation, as well as the evaluation of diagnostic performance. From an analytical perspective, the use of multiple platforms and laboratories is essential to assess the precision, accuracy, and reproducibility of biomarker measurements [46]. Clinically, the biomarker’s performance must be validated in independent patient cohorts to confirm its association with relevant clinical outcomes. In addition, key performance metrics such as sensitivity and specificity must be thoroughly evaluated [47].

## 6. Future Perspectives

Since the majority of AATD cases remain undiagnosed, these individuals, though patients in their own right, often do not receive treatment. Therefore, it makes sense to prioritize early diagnosis of the disease. Over the past decade, new testing methods have been developed, offering rapid, reliable alternatives to traditional approaches. One such test, the AAT Genotyping Test, is a minimally invasive technique that uses DNA extracted from a buccal swab or dried blood spot to simultaneously analyze the 14 most prevalent AATD mutations [48]. Now that LC-MS has become a standard platform in many clinical laboratories, the approach described above [21,42,43] could support/serve as a complementary method for this analysis. It is an important advancement as the potential of this technique to move beyond the lab will be crucial in bridging the gap between bench research and patient care. This will enable the swift translation of new discoveries into real-world clinical applications and could help address the issue of underdiagnosis thus increasing the detection rate of AATD and potentially reducing the harmful effects of delayed diagnosis. Another critical aspect that requires further development is the treatment of the disease. In AATD, diagnosis and treatment are closely connected, earlier diagnosis in fact offers access to better treatment options and, in turn, improves patient outcomes. AATD involves treating both lung and liver diseases, which are distinct pathologies. Augmentation therapy, the only specific treatment for AATD, has proven effective in treating lung disease but not in addressing the damaging polymers that cause liver disease and death in patients [19,49]. Thus, the assessment of liver fibrosis is crucial in the evaluation and follow-up of these patients. While liver transplantation can effectively treat AATD, it is not appropriate in the early stages of the disease. Several new therapeutic approaches are being explored for the treatment of AATD-related liver disease. These strategies aim to reduce the intrahepatic Z-AAT burden by reducing Z-AAT polymers through stimulation of their degradation or silencing their production and/or correcting or stabilizing the folding of the mutated Z-AAT to enable its secretion [49]. Silencing the production of mutated AAT using small-interfering RNAs shows promise as a strategy compared to other approaches [50]. DNA and RNA editing hold the potential to not only reduce the production of mutant Z-AAT but also increase the synthesis of wild-type M-AAT. This will, effectively address both the gain-of-function liver disease and the loss-of-function lung disease in affected individuals. Additionally, it enables the preservation of the body’s natural regulatory mechanisms for SERPINA1 expression, ensuring that wild-type M-AAT is produced as an acute phase reactant in response to inflammation and tissue injury [49]. Data from proteomic and metabolomic analyses could offer valuable insights for evaluating therapeutic responsiveness to potential drug. We are confident that the LC-MS platform could play a crucial role in analyzing the glycans present on Z-AAT in the future. This will offer a deeper understanding of how glycosylation affects the immune-modulatory functions of AAT. While extensive glycoanalysis has been conducted on normal AAT from healthy individuals, only one study has so far examined plasma AAT from Z-AAT-deficient individuals [51]. Nonetheless, the increased fucosylation observed on the N-glycans of Z-AAT in this study suggests ongoing inflammation in individuals with AATD, underscoring the potential for early therapeutic intervention.

One might question whether animal models could provide more detailed insights into the underlying mechanisms of the disease and guide future treatment strategies.

Given the limitations of mouse models in studying lung diseases, PiZZ ferret models have been used as a platform for preclinical testing of therapeutics, including gene therapy, for the progressive COPD seen in AAT-deficient patients [52]. Despite some differences between the ferret models of AATD and human disease, the similarities offer hope that this model could enable longitudinal assessment of pulmonary function in humans [52]. The expected answers from this approach aim to address several critical questions, such as the onset of the disease, its initial manifestations, and the ideal time for intervention. Additional inquiries focus on the roles of AAT beyond its antiprotease function, the most suitable genetic therapy for this complex gain- and loss-of-function disease, and whether gene therapy alone can reverse lung function decline after the disease has progressed. Clearly, proteomics and metabolomics analyses of fluids and tissues from these animals could offer significant insights in resolving these questions.

## 7. Conclusions

Two central questions frequently arise among physicians: Is AATD truly a rare disease? And is it a fatal inherited disorder, or treatable? The ongoing efforts in identifying disease biomarkers and developing therapies that complement or replace augmentation therapy appear to be aimed at shifting the long-held perception of the disease. Although the application of omics technologies in the study of AATD remains limited, the increasing use of integrated or multi-omics approaches, including transcriptomics, proteomics, and metabolomics, is expected to facilitate the identification of potential biomarkers. These biomarkers could play a crucial role in early diagnosis and support the development of targeted treatments through advances in therapeutic strategies. We are confident that the advancements achieved to date have brought us substantially closer to answering the question raised in the title of this article: that proteomics, on its own or, more effectively, when integrated with other omics techniques, could represent a decisive step toward addressing this complex clinical challenge.

## Figures and Tables

**Table 1:** Summary of proteomic studies in AATD.

Reference	Subjects Investigated *	Source	Proteomic Technique	Main Findings
[21]	P = 5	Serum	LC-ESI-Triple TOF-MS	MS can easily identify S/Z mutations by detecting mass differences between S/Z and non-S/non-Z peptides. Combining peptide pattern analysis with AAT quantification via immunoassay ensures an accurate assessment of deficiency alleles in most patients.
[27]	C = 5607 (COPD) P = 317 (GRADS = 133 QUANTUM-1 = 38 Birmingham = 146)	Plasma	SomaScan v4.0	Common plasma biomarkers have been identified in both AATD and COPD patients, along with proteins associated with emphysema. PiZZ patients also exhibited biomarkers related to DLCO and emphysema. Additionally, PiZZ individuals undergoing augmentation therapy showed near-normal AAT levels.
[29]	P = 31 (QUANTUM-1)	Serum	Multiplexed immunoassay	Proteome analyses revealed that C-reactive protein, adipocyte fatty acid-binding protein, and tissue plasminogen activator were linked to emphysema progression, highlighting them as potential therapeutic targets for COPD.
[30]	C = 7 P = 8	FFPE liver tissues	nanoLC-ESI-Q-Exactive hybrid quadrupole-Orbitrap-MS	Among the 65 proteins upregulated exclusively in adult PiZZ samples, protein disulfide isomerase A4 (PDIA4) emerged as a promising therapeutic target. Its inhibition by cysteamine reduced Z-aggregate formation, while its silencing decreased oxidative stress, a hallmark of AATD-related liver disease.
[31]	C = 43 (NS = 9 SM without COPD = 9 SM with COPD stage I–II= 8, SM with COPD stage III–IV = 8 IPF = 9) P = 8	Lung tissue	2D-DIGE MALDI-TOF/TOF	The proteome analyses showed increased transglutaminase 2 (TGM2) across all sample types, reinforcing its potential as a diagnostic and therapeutic target for AATD-associated COPD.
[32]	C = 37 (HC = 30 COPD = 7) P = 31 (AATD = 6 COPD-AATD = 25)	Neutrophils Plasma	LC-MS/MS	AAT augmentation therapy influences the neutrophil membrane proteome by altering the levels of membrane-associated proteins in circulating neutrophils of AATD-COPD patients.
[34]	C = 60 (NS = 25 SM = 20 COPD = 15) AATD = 23	EBC	ESI-LTQ-Orbitrap-MS SELDI-TOF	Several inflammatory cytokines, type I and II cytokeratins, two isoforms of surfactant protein A (SP-A), calgranulins A and B, and AAT have been identified in the COPD and AATD groups.
[35]	HC = 11 P = 11	EBC	NMR	The analyses revealed that pyruvate metabolism is the most prominently involved pathway, with most metabolites originating from pyruvate.

* HC = healthy controls; C = controls; P = patients. Abbreviations: α1-antitrypsin (AAT); α1-antitrypsin variant (PiZZ): α1-antitrypsin deficiency (AATD); Birmingham α-1 Antitrypsin registry cohort (Birmingham); chronic obstructive pulmonary disease (COPD); diffusion capacity of the lung for carbon monoxide (DLCO); electrospray ionization (ESI); enzyme-linked immunosorbent assay (ELISA); exhaled breath condensate (EBC); formalin-fixed paraffin-embedded (FEPE); α-1 antitrypsin deficiency and sarcoidosis (GRADS); idiopathic pulmonary fibrosis (IPF); liquid chromatography (LC); linear trap quadrupole (LTQ); mass spectrometry (MS); matrix-assisted laser desorption ionization (MALDI); nuclear magnetic resonance (NMR); nonsmokers (NS); quadrupole (Q); QUANTitative Chest Computed Tomography UnMasking emphysema progression in α1-Antitrypsin Deficiency (QUANTUM-1); sodium dodecyl sulphate-gel electrophoresis (SDS-PAGE); smokers (SM); surface enhanced laser desorption ionization (SELDI); surfactant protein A (SP-A); time of flight (TOF).

**Table 2:** Unmet clinical and research needs in AATD.

Category	Unmet Need	Rationale
Biomarkers	Pediatric biomarkers	Lack of validated biomarkers to enable early diagnosis in infants and young children.
	Prognostic biomarkers	Identification of markers that can predict disease progression and complications.
	Biomarkers to guide therapy	Lack of markers that inform therapeutic decision-making and response assessment
Standardization/Screening	Robust liver-disease correlates	Need for non-invasive, reliable markers that correlate with liver disease severity and progression.
	Pre-analytical protocols	Absence of standardized procedures for sample collection and handling in proteomic studies on AATD
	Broad implementation of screening strategies	Insufficient screening, particularly in asymptomatic individuals and family members

## Abbreviations

The following abbreviations are used in this manuscript:

AAT	α1-antitrypsin
AATD	α1-antitrypsin deficiency
COPD	Chronic obstructive pulmonary disease
IEF	Isoelectric focusing
LC-MS/MS	Liquid chromatography tandem mass spectrometry
DIGE	Two-dimensional difference gel electrophoresis
IPF	Idiopathic pulmonary fibrosis
NS	Non-smokers
HS	Healthy smokers
ELISA	Enzyme-linked immunosorbent assay
